# Emotional and Behavioral Outcomes in Childhood for Survivors of Invasive Group B *Streptococcus* Disease in Infancy: Findings From 5 Low- and Middle-Income Countries

**DOI:** 10.1093/cid/ciab821

**Published:** 2021-11-02

**Authors:** Jaya Chandna, Wan-Hsin Liu, Ziyaad Dangor, Shannon Leahy, Santhanam Sridhar, Hima B John, Humberto Mucasse, Quique Bassat, Azucena Bardaji, Amina Abubakar, Carophine Nasambu, Charles R Newton, Clara Sánchez Yanotti, Romina Libster, Kate Milner, Proma Paul, Joy E Lawn, GBS Low and Middle income Collaborative Group for Long-term Outcomes group, Shabir A Madhi, Z D, S L, Lois Harden, Azra Ghoor, Sibongile Mbatha, Sarah Lowick, Tamara Jaye, Sanjay G Lala, Pamela Sithole, Jacqueline Msayi, Ntombifuthi Kumalo, Tshepiso Nompumelelo Msibi, S S, H B J, Asha Arumugam, Nandhini Murugesan, Nandhini Rajendraprasad, Mohana Priya, A A, C N, Adam Mabrouk Adan, Patrick Vidzo Katana, Eva Mwangome, C R N, Q B, Azucena Bardají, Justina Bramugy, H M, Sergio Massora, R L, C S Y, Valeria Medina, Andrea Rojas, Daniel Amado, Conrado J Llapur, A K M

**Affiliations:** 1 Maternal, Adolescent, Repr oductive & Child Health Centre, London School of Hygiene & Tropical Medicine, London, United Kingdom; 2 Division of General Paediatrics, Department of Paediatrics, Taipei Veterans General Hospital, Taipei, Taiwan; 3 Department of Paediatrics and Child Health, Faculty of Health Sciences, University of the Witwatersrand, Johannesburg, South Africa; 4 Neonatology Department, Christian Medical College, Vellore, India; 5 Centro de Investigação em Saúde de Manhiça, Maputo, Mozambique; 6 ISGlobal, Hospital Clínic, Universitat de Barcelona, Barcelona, Spain; 7 Institució Catalana de Recerca i Estudis Avançats, Barcelona, Spain; 8 Pediatrics Department, Hospital Sant Joan de Déu (University of Barcelona), Barcelona, Spain; 9 Consorcio de Investigación Biomédica en Red de Epidemiología y Salud Pública, Madrid, Spain; 10 Neuroscience Research Group, Department of Clinical Sciences, KEMRI Wellcome Trust, Kilifi, Kenya; 11 Institute of Human Development, Aga Khan University, Nairobi, Kenya; 12 Department of Psychiatry, Medical Sciences Division, University of Oxford, Oxford, United Kingdom; 13 Fundación INFANT, Buenos Aires, Argentina; 14 Neurodisability & Rehabilitation Research Group, Murdoch Children’s Research Institute 2, Department of Paediatrics, University of Melbourne, Melbourne, Australia

**Keywords:** Group B Strep, neurodevelopment, neonatal sepsis, emotional behavior

## Abstract

**Background:**

Survivors of invasive group B *Streptococcus* (iGBS) disease, notably meningitis, are at increased risk of neurodevelopmental impairment. However, the limited studies to date have a median follow-up to 18 months and have mainly focused on moderate or severe neurodevelopmental impairment, with no previous studies on emotional-behavioral problems among iGBS survivors.

**Methods:**

In this multicountry, matched cohort study, we included children aged 18 months to 17 years with infant iGBS sepsis and meningitis from health demographic surveillance systems, or hospital records in Argentina, India, Kenya, Mozambique, and South Africa. Children without an iGBS history were matched to iGBS survivors for sex and age. Our primary outcomes were emotional-behavioral problems and psychopathological conditions as measured with the Child Behavior Checklist (CBCL). The CBCL was completed by the child’s primary caregiver.

**Results:**

Between October 2019 and April 2021, 573 children (mean age, 7.18 years) were assessed, including 156 iGBS survivors and 417 non-iGBS comparison children. On average, we observed more total problems and more anxiety, attention, and conduct problems for school-aged iGBS survivors compared with the non-iGBS group. No differences were found in the proportion of clinically significant psychopathological conditions defined by the *Diagnostic and Statistical Manual of Mental Disorders* (Fifth Edition).

**Conclusions:**

Our findings suggested that school-aged iGBS survivors experienced increased mild emotional behavioral problems that may affect children and families. At-risk neonates including iGBS survivors need long-term follow-up with integrated emotional-behavioral assessments and appropriate care. Scale-up will require simplified assessments that are free and culturally adapted.

**KEY FINDINGS**

1. What is known and what is new?

There is a paucity of comparable data regarding emotional-behavioral outcomes for early-middle childhood and school-age, especially from low- and middle-income countries. To our knowledge, there are no studies that use a standardized, validated emotional-behavioral assessment tool such as the Child Behavior Checklist (CBCL), which, though developed in the United States, is used widely. A multicountry study of long-term outcomes for infant survivors of invasive group B *Streptococcus* (iGBS) enabled us to address this gap.

2. What did we do and what did we find?

We used the CBCL, a caregiver-administered tool, to measure outcomes from 156 survivors of infant iGBS and 417 children without history of iGBS (non-iGBS group) from 5 countries across South Asia, sub-Saharan Africa, and Latin America. We found that school-aged iGBS survivors have more total emotional-behavioral problems than children in the non-iGBS group, and this difference seems to be mainly driven by internalizing problems (scales for emotional reactivity, anxiety/depression, withdrawal, and somatic problems). However, we detected no differences between iGBS survivors and children in the non-iGBS group for more severe clinical disorders defined as psychopathological conditions according to the *Diagnostic and Statistical Manual of Mental Disorders* (Fifth Edition). Importantly, we found major differences between countries, with one country recording no social emotional problems.

3. What now for programs?

Assessment of emotional-behavioral development is important for every child and all families and countries as part of the Sustainable Development Goals, enabling optimal human capital worldwide. Follow-up of at-risk neonates, including iGBS survivors, is needed and requires context-specific integration within child health programs and education settings.

4. What next for research?

CBCL and other developmental assessment tools require cultural adaptation to varying settings in order to accurately detect true differences. Importantly, there is currently no freely accessible and adaptable tool that is validated, which is a crucial challenge impeding uptake.

Despite the substantial potential impact of emotional-behavioral problems on children, their families, and human capital, there are limited data on the prevalence of emotional-behavioral problems, in particular those with milder or nonclinical problems. Mild neurodevelopmental impairments (NDIs), emotional-behavioral problems, and specific learning difficulties tend to become apparent later, especially once children transition to primary school without intervention in low-resource settings, these mild problems may affect educational attainment and the child’s life course [1, 2]. 

In the Global Burden of Disease Study, 2 mental health disorders, depression and anxiety, were among the top 10 causes of global disability-adjusted life-years in adolescents and young adults [3]. An estimated 52.9 million children younger than 5 years had developmental disabilities in 2016, and 95% were in low- and middle-income countries (LMICs). Estimates were generated for autism disorders and attention-deficit/hyperactivity disorder, but it was also noted there were very limited data, especially from Saharan Africa [4].

Measuring emotional-behavioral problems in preschool and school-aged children allows us to assess their executive functioning, which is predictive of long-term adult functioning. Internalizing behavioral problems (such as anxiety or depression) by their nature may be more challenging to detect without active surveillance, even in school-aged children, whereas externalizing behaviors (eg, rule-breaking and aggressive behavior), which are typically disruptive to others, may be more readily detected by parents or teachers [5, 6].

Group B *Streptococcus* (GBS) is a leading pathogen causing neonatal and young infant infection [7, 8], notably as sepsis, meningitis, or pneumonia, collectively known as invasive GBS (iGBS) disease [7, 9]. Some studies have reported emotional-behavioral difficulties in about 10% to one-third of bacterial meningitis survivors, which can significantly impair their quality of life and activities of daily living [2, 10]. The only systematic review of NDI after iGBS reported results after meningitis, with a median follow-up of 18 months, and focused on intellectual, motor, vision, and hearing impairment [11].

Major neurological sequelae, such as epilepsy, mental retardation, cerebral palsy, and moderate or severe sensory impairments, are more readily identifiable in early life (age 0–3 years) [11]. None of the studies specifically included emotional-behavioral problems. A multicountry study [9], investigating the long-term outcomes for iGBS survivors provided an opportunity to evaluate the prevalence of emotional-behavioral problems among children from toddlers to the end of secondary school.

This article is part of a series on GBS worldwide. The aim of the study was to estimate the prevalence of emotional-behavioral problems in iGBS survivors aged 18 months to 17 years, using data collected in Argentina, India, Kenya, Mozambique, and South Africa. The objectives were to (1) describe characteristics of iGBS survivors and the non-iGBS comparison group; (2) assess differences in emotional-behavioral problems between iGBS survivors and the non-iGBS comparison group, using the CBCL Syndrome Scale and *Diagnostic and Statistical Manual of Mental Disorders* (Fifth Edition) (*DSM*–*5*) categories; and (3) compare the prevalence of clinical range problems using the clinical cutoffs for the syndrome scale between iGBS survivors and the non-iGBS comparison group

## METHODS

### Overall Study Design, Setting, and Case Definitions

This work was part of a matched, multicountry cohort study to estimate the long-term health outcomes and economic costs for children surviving iGBS. Information regarding the research protocol and methods have been published [9]. In summary, data were collected from 5 LMICs: Kenya, Mozambique and South Africa (Africa), India (Asia), and Argentina (Latin America). Children exposed to neonatal or infant iGBS disease were identified through hospital admission records in Argentina, India, Kenya, and South Africa and by surveillance of laboratory services in Mozambique and South Africa. All children who met the case definitions of iGBS were approached. Children without a history of iGBS disease were recruited via hospital network in Argentina, India, and South Africa or using the health and demographic surveillance systems in Kenya and Mozambique and matched to iGBS survivors for sex and age at a 3:1 ratio; more details can be found elsewhere for individual country recruitment [13–15]. The sample size was based on detection of a 16% difference in severe or moderate NDIs, which was inclusive of emotional-behavior outcomes; a detailed power calculation is described elsewhere [9, 12–15].

Case definitions for iGBS included clinical signs of possible serious bacterial infection and detection of GBS from a normally sterile site in an infant <90 days old. iGBS was further categorized according to disease onset and clinical syndrome. Early-onset disease was defined as disease occurring in infants aged 0–6 days, and late-onset disease as those in infants aged 7–89 days. There are 2 main clinical syndromes of iGBS disease: GBS meningitis and sepsis (Supplementary Table 2).

Trained fieldworkers scheduled a one-time health facility visit with the enrolled children and their parents or main caregivers. At the assessment visit, the parents or main caregivers provided information on health, demographic and economic characteristics, and health-related quality of life [9]. Children were assessed with a set of neurodevelopmental assessments that were age and culturally appropriate [9]. All data were captured using paper forms or a tablet-based, customized application.

### Assessment of Social Emotional Problems Using the Child Behavior Checklist

Across all 5 study sites, the emotional and behavior outcomes were assessed using the Child Behavior Checklist (CBCL). The CBCL, developed by the Achenbach System of Empirically Based Assessment (ASEBA), is a self-administered questionnaire completed by caregivers to identify a broad spectrum of adaptive functioning and problems. The checklist has 2 versions. For children ≤6 years old, the CBCL/1.5–5 version was used, and the CBCL/6–12 version was applied to children >6 years old [16, 17]. The instrument has good reliability (test-retest correlation, 0.90; Cronbach α = 0.92) and has been widely validated in numerous cultural and geographic settings [18, 19]. The study staff who gave the questionnaire to the parents were not blinded to the iGBS exposure status of the children.

The CBCL obtains parents’ responses based on observation of their children’s behavior in the preceding 6 months. Following the instructions in ASEBA, similar items were grouped together and their scores were added up to provide raw scales for syndromes. CBCL/1.5–5 and CBCL/6–18 measure different problem scales (1.5–5 years: emotional reactivity, anxiety/depression, withdrawal, somatic problems, sleep problems, attention problems, and aggressive behavior; 6–18 years: anxiety, depression, somatic problems, social problems, thought problems, attention problems, rule-breaking and aggressive behavior), but yield results in common scales, the internalizing and externalizing problem scales. The total problem score is the sum of all problem items. CBCL also produces *DSM-5*–oriented scales, which are derived from experts’ consensus on the items’ consistency with the diagnostic criteria for *DSM-5* disorders (Supplementary Table 3) [20]. Scores were not calculated at the time of the assessment; however, children were referred for support based on a comprehensive developmental assessment carried out by a trained clinician.

To ensure comparability between studies, raw scores were converted into age- and sex- standardized T scores, using the conversion tables provided by the ASEBA. For each scale, its T score can be interpreted as falling within the normal, borderline, or clinical range. The clinical range is grouped in accordance with ASEBA guidance to avoid false-negatives, and children whose scales fall into that ranges are of clinical significance and are suggested to receive clinical consultation [20]. For problem scales, T scores ≥60 are classified as borderline/clinical, and T scores <60 as normal, while the cutoff point for *DSM-5*–oriented scales is 65 [17, 20]. Data sharing and transfer agreements were jointly developed and signed by all collaborating partners; data will be available on request.

### Ethical Consideration and Consent to Participate

The overarching protocol for this multicountry observational study was granted ethical approval at the London School of Hygiene & Tropical Medicine (approval no. 16246). Institutional review boards in each of the operating countries granted ethical approval (Argentina, protocol EGB-1; India, approval nos. 11723 [Christian Medical College, Vellore] and 2019-7034 [Indian Council of Medical Research]); Kenya, approval no. SERU/CGMR-C/164/3882; Mozambique, approval no. 98/CNBS/2019; South Africa, approval no. M190241), along with the institutional review board of the World Health Organization (approval no. ERC0.0003169). Written informed consent is obtained from parents or guardians, and when appropriate, based on local guidelines, assent is also obtained from participating children.

### Statistical Analysis

Descriptive analyses were performed to compare baseline characteristics between iGBS survivors and children in the non-iGBS group; we used Pearson’s χ ^2^ tests for categorical and 2-sample *t* tests for continuous variables. We used linear regression to assess differences in the raw problem scores between groups, adjusting for study site and matching age and sex variables when pooling the data. We also conducted sensitivity analyses, excluding children with moderate to severe NDIs. All analyses were conducted using Stata software, version 15.1. 

## RESULTS

### Overall

**Figure 1.**
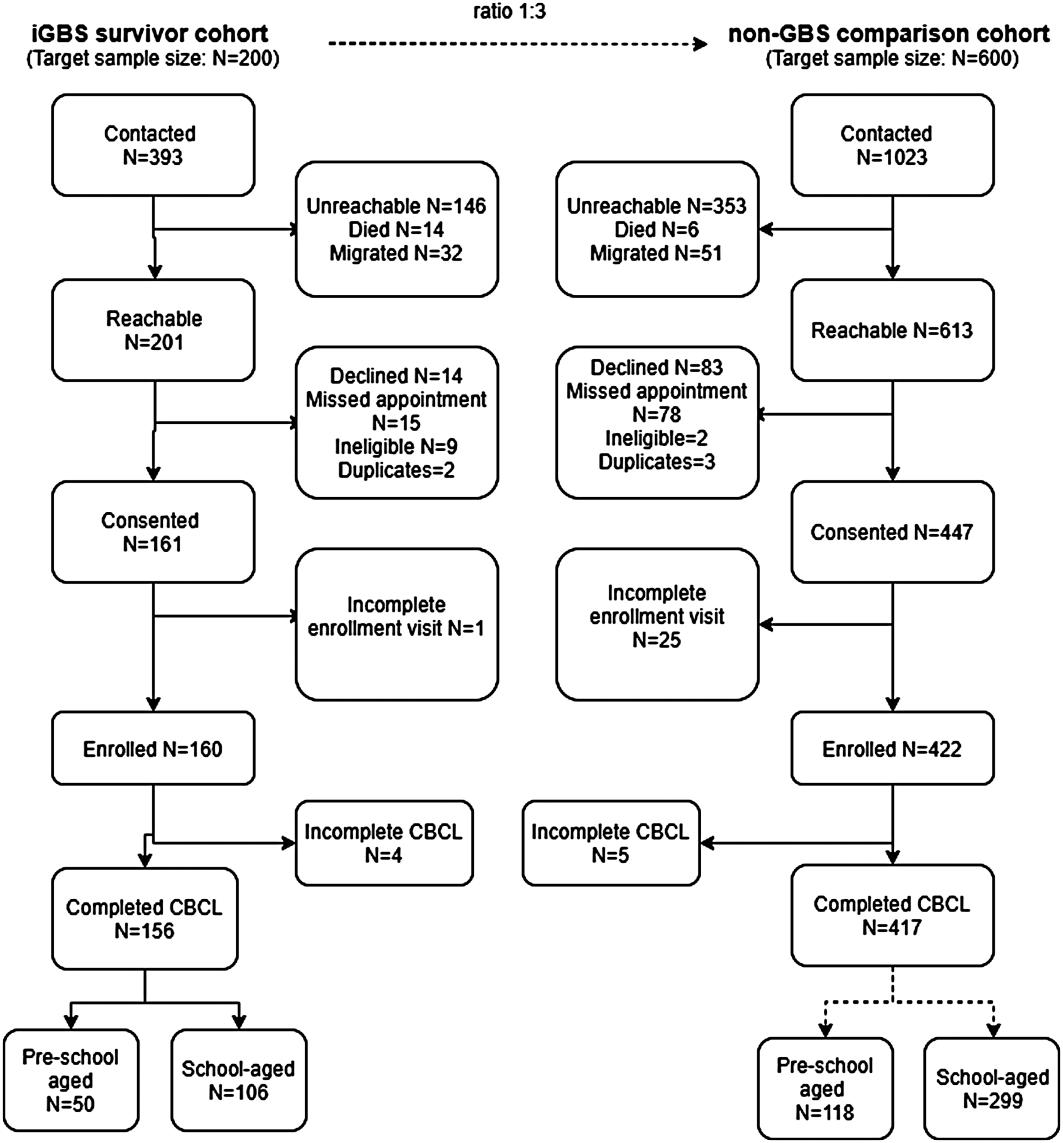
Participant flow of children with or without invasive group B *Streptococcus* (iGBS) recruited to the study. Of 393 iGBS survivors contacted, 160 consented to participation and completed the assessment. Of 1023 matched children in the non-iGBS group contacted for participation, 422 consented and completed neurodevelopmental, vision, and hearing assessments. Abbreviation: CBCL, Child Behavior Checklist.

Between October 2019 and April 2021, iGBS survivors and nonsurvivors were enrolled in a multicountry matched cohort study from Argentina, India, Kenya, Mozambique, and Kenya. Of the 393 iGBS survivors and 1023 in the non-iGBS group initially contacted, we failed to approach approximately a third of eligible children in both cohorts. A total of 156 survivors (39.7%) and 417 (comparison children 40.8%) were enrolled and completed the CBCL (Figure 1). Among the 573 children with CBCL assessment, 405 (70.7%) of the cohort completed the school-aged (>6 years old) assessment tool, and the remaining 168 (29.3%) completed the preschool-aged (≤6 years old) assessment (Figure 1). India and Kenya made up 73.8% of the preschool cohort, while Mozambique and South Africa accounted for 26.6% of the school-aged cohort population. Argentina made up <5% of the total cohort (Table 1).

**Table 1.** Descriptive Characteristics of Invasive Group B *Streptococcus* (iGBS) Survivors and Non-iGBS Comparison Group From India, South Africa, Mozambique, Kenya, and Argentina

	Preschool-Aged Cohort (≤6 y), No. (%) (n = 168)		*P* Value	School-Aged Cohort (>6 y), No. (%) (n = 405)		
Characteristic	iGBS Survivors (n = 50)	Non-iGBS Group (n = 118)	*P* Value	iGBS Survivors (n = 106)	Non-iGBS Group (n = 299)	*P* Value
Country						
India	23 (46.0)	44 (37.3)	.51	10 (9.4)	17 (5.7)	.01
Kenya	13 (26.0)	44 (37.3)		16 (15.1)	64 (21.4)	
Mozambique	8 (16.0)	22 (18.6)		31 (29.2)	100 (33.4)	
South Africa	4 (8.0)	5 (4.2)		39 (36.8)	112 (37.5)	
Argentina	2 (4.0)	3 (2.5)		10 (9.4)	6 (2.0)	
Female sex	28 (56.0)	66 (55.9)	.99	45 (42.5)	149 (49.8)	.16
Preterm	7/47 (14.9)	7/117 (6.0)	.15	11/96 (11.5)	22/286 (7.7)	.31
Low birthweight	10 (20.0)	16/109 (14.7)	.40	20/95 (21.1)	31/214 (14.5)	.15
Birth order						
1st	31 (62.0)	41 (34.8)	.003	42 (39.6)	106 (35.5)	.09
2nd	7 (14.0)	38 (32.2)		31 (29.3)	65 (21.7)	
3rd or higher	12 (24.0)	39 (33.0)		33 (31.1)	128 (42.8)	
Main caregiver’s educational attainment						
College or university	35 (70.0)	92 (78.0)	.27	88 (83.0)	255 (85.3)	.58
High school or below	15 (30.0)	26 (22.0)		18 (17.0)	44 (14.7)	

Abbreviation: iGBS, invasive group B *Streptococcus*.

### Objective 1: Characteristics Among iGBS Survivors and the Non-iGBS Comparison Group

There were no differences between iGBS and non-iGBS cohorts for age, sex, and the main caregiver’s educational attainment in each country. We observed that preschool-aged iGBS survivors were more likely born preterm (14.9% vs 6.0%), but the differences didn’t reach statistical significance (*P* = .15). The trend could also be seen in the school-aged cohort (11.5% vs 7.7%; *P* = .31) (Table 1).

### Objective 2: Emotional-Behavioral Problems: Syndrome Scale and *DSM-5* Scale

In the school-aged cohort, we observed that the iGBS survivors had significantly higher total emotional behavioral problem scores than children in the non-iGBS group (adjusted mean difference, 3.85; 95% confidence interval, .33–7.38; *P* = .03). These scores were driven by both the internalizing (subscales for anxiety and withdrawal) and externalizing (aggressive syndrome subscale) scores (Table 2). Conversely, in the preschool-aged cohort, we detected no differences in emotional-behavioral problem scores between the iGBS survivors and the non-iGBS comparison cohort (Table 2). The results were similar when removing children with moderate or severe NDI (Supplementary Table 7). Scores for total, externalizing, and internalizing problems were similar between countries, except for Mozambique, which had the lowest scores in all 3 categories, and Argentina, which had the highest external problem score (Supplementary Table 4).

**Table 2.** Mean Differences in Child Behavior Checklist Problem Scores, Stratified by Age Cohort, From October 2019 to April 2021

	Mean CBCL Score^a^			
Problems by Age Cohort	iGBS Survivors	Non-iGBS Group	Adjusted Mean Difference (95% CI)^b^	*P* Value
Preschool-aged cohort (≤6 y)	n = 50	n = 118		
Total problems	21.48	20.90	−0.45 (−6.59 to 5.68)	.88
Externalizing problems	8.08	7.32	0.01 (2.54 to 2.57)	.99
Attention problems	1.46	1.73	−0.42 (−1.07 to .23)	.20
Aggressive behavior	6.62	5.59	0.44 (−1.65 to 2.52)	.68
Internalizing problems	6.10	6.21	−0.13 (−2.03 to 1.78)	.90
Emotional reactivity	1.18	1.22	−0.06 (−.67 to .55)	.84
Anxiety/depression	1.68	2.03	−0.42 (−1.03 to .19)	.17
Withdrawal	1.08	1.49	−0.33 (−1.00 to .35)	.34
Somatic problems	2.16	1.47		
School-aged cohort (>6 y)	n = 106	n = 299		
Total problems	23.49	17.51	3.85 (.33 to 7.38)	.03
Externalizing problems	5.82	4.29	0.97 (−.06 to 2.00)	.06
Rule-breaking behavior	1.75	1.25	0.36 (−.002 to .73)	.051
Aggressive behavior	4.08	3.04	0.61 (−.15 to 1.37)	.12
Internalizing problems	6.78	5.11	1.14 (.02 to 2.27)	.046
Anxiety/depression	3.12	2.39	0.45 (−.12 to 1.02)	.12
Withdrawal/depression	1.78	1.19	0.46 (.05 to .86)	.03
Somatic problems	1.88	1.53	0.23 (−.24 to .71)	.33

Abbreviations: CBCL, Child Behavior Checklist; CI, confidence interval; iGBS, invasive group B *Streptococcus*.

^a^Unadjusted mean scores.

^b^Adjusted for study site, age, and sex.

For school-aged children, the *DSM-5* categorization showed that iGBS survivors had higher scores for anxiety (adjusted mean difference, 0.50; *P* = .02), attention (0.64; *P* = .01) and conduct (0.44; *P* = .06) problems, after adjustment for age, sex, and study site. Conversely the preschool-aged children showed no differences between iGBS survivors and the non-iGBS comparison group (Table 3).

**Table 3.** Mean Differences in *DSM-5*–Oriented Scores by Age Cohort, From October 2019 to April 2021

	Mean *DSM-5*-Oriented Score			
Problems by Age Cohort	iGBS Survivors^a^	Non-iGBS Group^a^	Adjusted Mean Difference^b^ (95% CI)	*P* Value
Preschool-aged cohort (≤6 y)	n = 50	n = 118		
Depressive problem	1.14 (.64–1.64)	1.06 (.77–1.35)	0.01 (−.54 to .56)	.97
Anxiety problem	2.28 (1.67–2.89)	2.50 (2.03–2.97)	−0.32 (−1.08 to .45)	.41
Attention problem	2.64 (1.92–3.36)	2.47 (2.00–2.95)	0.03 (−.76 to .82)	.95
Oppositional problem	2.26 (1.52–2.99)	1.91 (1.43–2.38)	0.12 (−.68 to .92)	.76
Autism problem	2.08 (1.23–2.87)	1.93 (1.45–2.41)	0.14 (−.72 to 1.00)	.75
School-aged cohort (>6 y)	n = 106	n = 299		
Depressive problem	2.02 (1.60–2.44)	1.45 (1.20–1.70)	0.34 (−.09 to .77)	.12
Anxiety problem	2.52 (2.09–2.95)	1.78 (1.51–2.04)	0.50 (.07 to .93)	.02
Somatic problem	1.16 (.82–1.50)	1.14 (.95–1.33)	0.03 (−.33 to .39)	.88
Attention problem	3.09 (2.52–3.66)	2.14 (1.83–2.45)	0.64 (.14 to 1.14)	.01
Oppositional problem	1.48 (1.13–1.83)	1.16 (.97–1.35)	0.10 (−.23 to .42)	.56
Conduct problem	1.96 (1.53–2.40)	1.40 (1.15–1.66)	0.44 (−.01 to .90)	.057

Abbreviations: CI, confidence interval; *DSM-5, Diagnostic and Statistical Manual of Mental Disorders* (Fifth Edition); iGBS, invasive group B *Streptococcus*.

^a^Unadjusted mean scores.

^b^Adjusted for study site, age and sex.

### Objective 3: Emotional- Behavioral Problems in Clinical Range

There were no significant differences in the proportion of children in either age group’s CBCL scores in the clinical range in the iGBS and non-iGBS comparison cohort (preschool children, *P* = .36; school-aged children, *P* = .51) (Supplementary Table 5). However, when comparing countries, there was some variability between sites; of note, no clinically significant problems were detected in Mozambique in either the iGBS group or the non-iGBS comparison group (Supplementary Tables 6*A* and 6*B*).

## Discussion

Our multicountry study of long-term outcomes provides the first data on emotional-behavioral problems in preschool- and school-aged groups, using a standard tool and training across 5 LMICs and, importantly, with locally selected, matched children as a counterfactual. We were able to compare results between countries and also between iGBS survivors and children in the non-iGBS group, as well as following iGBS sepsis, which has not been reported before from LMICs.

Among school-aged children, iGBS survivors were detected as having increased numbers of total problems (in both externalizing and internalizing scales) and higher scores for some *DSM-5* psychopathological conditions, including anxiety, attention, and conduct disorders. These findings align with those of 2 case-control studies after meningococcal meningitis, which found that the school-aged survivors had significantly more anxiety, conduct, and attention problems than their controls [12, 21]. Other studies reported that school-aged survivors of neonatal or childhood bacterial meningitis were more likely than healthy controls to be rated by their parents as having behavioral problems [22, 23]. A systematic review suggested that adult sepsis survivors had an increased risk of long-term cognitive problems [24]. However, we also found 2 studies that refuted the increased behavioral problems in children after meningitis [1, 25]. Notably, all of these studies were based in high-income countries and so may not be comparable to the contexts of our study.

Conversely, in preschool children we found no differences in total number of problems, problems within a clinical range, or *DSM-5*–oriented scores. It is recognized that at this age, social-emotional problems are more difficult to detect and may not identified by parent report scales [26]. In addition, as we detected no significant differences in scores within the clinical range in either age group, it is plausible that mild social-emotional problems are apparent only later in childhood and therefore differences were detected only in the school-aged group.

Interestingly we found some variation in total problems and clinical level problems between countries, with Mozambique recording zero clinical range problems in either group and a very low number of total problems (Supplementary Tables 6*A* and 6*B*). Hence, cultural variation in measurement and reporting may be a challenge, even when using the same CBCL tool [27].

A major strength of our study was the multicountry design and the use of the same CBCL tool across all countries, including use of a software app and standard training for all sites. The use of a local, matched counterfactual group is also a strength, rather than comparison with normative standards, as is usual in developmental data analysis. Although the sample size was small, this is still a relatively large cohort compared with previous studies of emotional-behavioral problems, and it includes data collected from a range of settings.

However, our study highlights a weakness of the tool. Although the CBCL is well validated in a number of settings, the adaptation of constructs is often overlooked in the validation process [28]. Hence, CBCL may still not be appropriate in all settings, as evidenced in Mozambique, where no clinical problems were detected.

A limitation of our study is that though it is larger than previous studies, the sample size is small. The coronavirus disease 2019 pandemic posed a challenge to recruitment, which resulted in lower power to detect differences between the iGBS and non-iGBS groups. Another limitation was the low participation rates. Approximately a third of eligible participants could not be contacted, and this could lead to selection bias. We did not adjust for preterm or low birthweight, since across most countries there were no differences between the iGBS and non-iGBS groups, though there is a nonsignificant trend that toward more preterm births among iGBS survivors. However, we did adjust for study site. There was a lack of coexposure data on neonatal risks, such as multiple birth and hypoxic encephalopathy, which meant we could not adjust for possible confounding.

Our findings show the importance of longer-term follow-up to school age, when emotional-behavioral problems are likely to become apparent, to prevent dual disadvantage due to potential impact on educational engagement and performance. However, more population data are needed to interpret the lack of difference in clinically significant problems between groups and the unexpectedly low prevalence of problems in Mozambique. Qualitative exploration of the potential role of cultural differences in parental reporting across settings is warranted.

Implementation research is needed to develop context-specific approaches to integrate testing for at-risk newborns and children, including iGBS survivors, into routine child health programs, and to provide health and educational support if needed. Because most problems were detected at school age, families and teachers should be made more aware of possible emotional-behavioral difficulties and the impact these can have on school performance if not adequately addressed. Importantly, behavioral assessment tools are needed that are open access and can be used across cultures in a standardized way.

Our findings contributed new evidence on the risk of long-term emotion-behavioral problems after iGBS. As well as important implications for monitoring children, these findings have implications for the higher potential impact of prevention of GBS, including possible gains from a GBS maternal vaccine.

In conclusion, these findings have implications for both programs and research. Follow-up of at-risk neonates, including iGBS survivors, is needed and requires context-specific integration within child health programs. There is a need for culturally sensitive detection tools that carefully consider how constructs of social-emotional behavior can be measured in different settings. To achieve equity of measurement, these tools must be open access, free, and usable by a wide ranges of workers [29]. Implementation research is needed to identify context-specific integration of social-emotional follow-up into routine child health programs. Assessment of emotional-behavioral development is important for every child and every family in every country as part of the Sustainable Development Goals, enabling optimal human capital worldwide.

## Supplementary Material

ciab821_suppl_Supplementary_Materials

## Notes

***Acknowledgments*.** First and most importantly, we thank the women and children participating in the group B *Streptococcus* (GBS) study and their families, without whom this work would not have been possible. We thank all the study staff for their hard work and dedication. Appreciation to Claudia da Silva for her administrative support.

***GBS Low and Middle income Collaborative Group for Long-term Outcomes group members.*** From the Faculty of Health Sciences, University of the Witwatersrand, Johannesburg, South Africa: Shabir A. Madhi (Medical Research Council: Vaccines and Infectious Diseases Analytical Unit and Department of Science and Technology/National Research Foundation: Vaccine Preventable Diseases), Z. D. (Medical Research Council: Vaccines and Infectious Diseases Analytical Unit), S. L. (Department of Paediatrics and Child Health), Lois Harden (Brain Function Research Group, School of Physiology), Azra Ghoor (Department of Paediatrics and Child Health), Sibongile Mbatha (Department of Paediatrics and Child Health), Sarah Lowick (Department of Paediatrics and Child Health), Tamara Jaye (Department of Paediatrics and Child Health), Sanjay G Lala (Department of Paediatrics and Child Health), Pamela Sithole (Medical Research Council: Vaccines and Infectious Diseases Analytical Unit), Jacqueline Msayi (Medical Research Council: Vaccines and Infectious Diseases Analytical Unit,), Ntombifuthi Kumalo (Medical Research Council: Vaccines and Infectious Diseases Analytical Unit), and Tshepiso Nompumelelo Msibi (Medical Research Council: Vaccines and Infectious Diseases Analytical Unit). From the Department of Neonatology, Christian Medical College, Vellore, India: S. S., H. B. J., Asha Arumugam, Nandhini Murugesan, Nandhini Rajendraprasad, and Mohana Priya. From KEMRI Wellcome Trust, Kilifi, Kenya: A. A. (Neuroscience Research Group, Department of Clinical Sciences; also from the Institute for Human Development, Aga Khan University, Nairobi, Kenya), C. N. (Neuroscience Research Group, Department of Clinical Sciences), Adam Mabrouk Adan, Patrick Vidzo Katana, Eva Mwangome, and C. R. N. (also from the Department of Psychiatry, Medical Sciences Division, University of Oxford, Oxford, United Kingdom). From Centro de Investigação em Saúde de Manhiça, Maputo, Mozambique: Q. B. (also from ISGlobal, Hospital Clínic, Universitat de Barcelona, Institució Catalana de Recerca i Estudis Avançats, and Pediatrics Department, Hospital Sant Joan de Déu [University of Barcelona], Barcelona, Spain; and Consorcio de Investigación Biomédicaen Red de Epidemiología y Salud Pública, Madrid, Spain), Azucena Bardají (also from ISGlobal, Hospital Clínic, Universitat de Barcelona), Justina Bramugy, H. M., and Sergio Massora. From ISGlobal, Hospital Clínic, Universitat de Barcelona: Celine Aerts. From Fundación INFANT, Buenos Aires, Argentina: R. L. and C. S. Y. From the Instituto de Maternidad y Ginecología Nuestra Señora de las Mercedes, Argentina: Valeria Medina, Andrea Rojas, Daniel Amado, and Conrado J. Llapur. From the Maternal and Child Health Division, International Centre for Diarrhoeal Disease Research, Bangladesh (icddr,b), Dhaka, Bangladesh: A. K. M. Tanvir Hossain and Qazi Sadeq-ur Rahman.

***Supplement sponsorship.*** This supplement is sponsored by Bill & Melinda Gates Foundation.

***Author contributions.*** The GBS study was conceptualized by J. E. L. The collaborative group contributed to the design of the study protocol and undertook data collection. For this article, J. C. , with W. H. L., P. P., and J. E. L., developed the research questions and the overall analysis plan and drafted the manuscript; these were refined with inputs from the wider collaborative group at a series of virtual workshops. W. H. L. performed analysis. P. P. provided statistical oversight. All authors reviewed and helped revise the manuscript and reviewed and agreed on the final version.

***Disclaimer.*** The funders had no role in study design, data collection and analysis, the decision to publish, or the preparation of the manuscript.

***Financial support.*** This work was supported by a grant (INV-009018) from the Bill & Melinda Gates Foundation to the London School of Hygiene and Tropical Medicine (principal investigator, J. E. L.).

***Potential conflicts of interest.*** Z. D. and S. L. report subawards from the London School of Hygiene & Tropical Medicine (LSHTM) to the Wits Health Consortium. S. S. reports subawards from LSHTM to Christian Medical College, Vellore. H. B. J. reports subawards from LSHTM to India. H. M. reports subawards from LSHTM to Mozambique. Q. B. reports subawards from LSHTM to ISGlobal and Centro de Investigação em Saúde de Manhiça/FM, Mozambique. A. B. reports subawards from LSHTM to Mozambique and funding from European & Developing Countries Clinical Trials Partnership (fellowship no. 2836; principal investigator Marieke Van der Zalm) outside the conduct of the study. A. A. reports subawards from the LSHTM to the University of Oxford. C. N. reports subawards from LSHTM to University of Oxford. C. R. N. reports subawards from LSHTM to Kenya Medical Research Institute, Kilifi, Kenya. C. S. Y. reports subawards from LSHTM to Fundación INFANT. R. L. reports a grant from Program for Appropriate Technology in Health for a respiratory syncytial virus (RSV) costing study, grants to Fernando Polack from the Bill & Melinda Gates Foundation for estimating the RSV burden of disease, consulting fees and participating for serving on Pfizer’s GBS advisory board and Janssen’s RSV advisory board, payment or honoraria for Janssen’s RSV lecture and Merck’s human papillomavirus lecture, and stock or stock options from iTRIALS. All other authors report no potential conflicts. All authors have submitted the ICMJE Form for Disclosure of Potential Conflicts of Interest. Conflicts that the editors consider relevant to the content of the manuscript have been disclosed.
