# Supplementary material for: Emotional and Behavioral Outcomes in Childhood for Survivors of Invasive Group B Streptococcus Disease in Infancy: Findings From 5 Low- and Middle-Income Countries
Source: Clin Infect Dis. 2021 Nov 2;74(Suppl 1):S35–43. doi: 10.1093/cid/ciab821 (PMC8776308; doi:10.1093/cid/ciab821)
Supplement: ciab821_suppl_Supplementary_Materials [file ciab821_suppl_supplementary_materials.docx]

**SUPPLEMENTARY MATERIAL**

**Supplement Title**: Every Country, Every Woman, Every Child; Group B Streptococcal Disease Worldwide

**Paper Title**: Emotional and behavioural outcomes in childhood for survivors of Group B Streptococcus invasive disease in infancy: findings from five low- and middle-income countries

**Short title**: Emotional and behavioral outcomes after iGBS

**AUTHORS:**

Jaya Chandna^1*^, Wan-Hsin Liu^1,2*^, Ziyaad Dangor^3^, Shannon Leahy^3^, Santhanam Sridhar^4^, Hima B John^4^, Humberto Mucasse^5^, Quique Bassat^5,6,7,8,9^, Azucena Bardaji^5,6^, Amina Abubakar^10,11^, Carophine Nasambu^10^, Charles R Newton^10,12^, Clara Sánchez Yanotti^13^, Romina Libster^13^, Kate Milner^14^, Proma Paul^1,+^ Joy E Lawn^1+^ on behalf of the LMIC collaborative group.

1. Maternal, Adolescent, Reproductive & Child Health (MARCH) Centre, London School of Hygiene & Tropical Medicine, London, UK
2. Division of General Paediatrics, Department of Paediatrics, Taipei Veterans General Hospital, Taipei, Taiwan
3. Department of Paediatrics and Child Health, Faculty of Health Sciences, University of the Witwatersrand, Johannesburg, South Africa
4. Neonatology Department, Christian Medical College, Vellore, India
5. Centro de Investigação em Saúde de Manhiça (CISM), Maputo, Mozambique
6. ISGlobal, Hospital Clínic, Universitat de Barcelona, Barcelona, Spain
7. ICREA, Pg. Lluís Companys 23, 08010 Barcelona, Spain
8. Pediatrics Department, Hospital Sant Joan de Déu (University of Barcelona), Barcelona, Spain
9. Consorcio de Investigación Biomédica en Red de Epidemiología y Salud Pública (CIBERESP), Madrid, Spain
10. Neuroscience Research Group, Department of Clinical Sciences, KEMRI-Wellcome Trust, Kilifi, Kenya
11. Institute of Human Development, Aga Khan University, Nairobi, Kenya
12. Department of Psychiatry, Medical Sciences Division, University of Oxford, Oxford, UK
13. Fundación INFANT, Buenos Aires, Argentina
14. Neurodisability & Rehabilitation Research Group, Murdoch Children’s Research Institute 2. Department of Paediatrics, University of Melbourne

* Authors share joint first authorship

+ Authors share senior authorship

Corresponding Author: Jaya Chandna, London School of Hygiene and Tropical Medicine, Keppel Street, London, WC1E 7HT. @: [jaya.chandna@lshtm.ac.uk](mailto:jaya.chandna@lshtm.ac.uk) T: [020 7636 8636](https://www.google.com/search?client=safari&rls=en&q=london+schol+of+hygiene&ie=UTF-8&oe=UTF-8)

Table of Contents

[Table of Contents 2](#_Toc81573471)

[Supplementary tables 3](#_Toc81573472)

[Supplementary Table 1. Sources of study population from the 5 countries 3](#_Toc81573473)

[Supplementary Table 2. Group B streptococcus syndrome definitions 4](#_Toc81573474)

[Supplementary Table 3. Assessment tool Child Behaviour Checklist and applicable measurement scales 5](#_Toc81573475)

[Supplementary Table 4. Adjusted means for iGBS survivors and non-GBS group (N=553) 6](#_Toc81573476)

[Supplementary Table 5. Proportion of clinically significant Child Behaviour Checklist problems in the preschool-aged and school-aged cohorts across five LMIC 7](#_Toc81573477)

[Supplementary Table 6A. Proportion of clinically significant Child Behaviour Checklist problems in the preschool-aged cohort in five LMIC 8](#_Toc81573478)

[Supplementary Table 6B. Proportion of clinically significant Child Behaviour Checklist problems in the school-aged cohort in five LMIC 8](#_Toc81573479)

[Supplementary Table 7. Sensitivity analysis: Mean differences of the CBCL problem scores, stratified by preschool-aged and school-aged cohort, excluding moderate and severe neurodevelopmental impairment, N=502 9](#_Toc81573480)

[STROBE Statement—Checklist of items that should be included in reports of cohort studies 11](#_Toc81573481)

# Supplementary tables

## Supplementary Table 1. Sources of study population from the 5 countries

| Country | Colloaborative institution | Identification of GBS-exposed children | | Identification of GBS-exposed children | Age at enrolment |
| --- | --- | --- | --- | --- | --- |
|  |  | Facilicty and location | Source and time interval |  |  |
| Argentina | Fundación Infant and Buenos Aires | 2 Public hospitals in Tucuman | Admitted neonates from 2003– 2016 | Primary Care Centers that belong to the Maternity Network | 3–16 years |
| India | Christian Medical College | Academic and referral hospital at Vellore | Hospital- delivered neonates from 2004–2018 | Hospital birth registry | 18 months – 15 years |
| Kenya | KEMRI- Wellcome Trust | Kilifi County Hospital | Admitted neonates from 2007–2018 | HDSS in Mbita and Kwale district | 1–12 years |
| Mozambique | Barcelona Institute for Global Health, Manhiça Health Research Centre | Manhiça District Hospital | Laboratory isolates conducted from 2001–2018 | HDSS in Manhica district | 3–17 years |
| South Africa | Wits Health Consortium | 3 Academic hospital in Johannesburg | Surveillance of pediatric wards and microbiology services from 2012– 2015 | Hospital medical registry | 5–7 years |

Modified from the protocol paper

Abbreviation: HDSS = Health Demographic Surveillance System

## Supplementary Table 2. Group B streptococcus syndrome definitions

| GBS syndrome | Definition |
| --- | --- |
| pSBI | Any one of the following: a history of difficulty feeding, history of convulsions, movement only when stimulated, respiratory rate of ≥ 60 breaths/min, severe chest retraction, temperature ≥ 37.5°C or ≤35.5°C. |
| Sepsis | Clinical signs of pSBI and isolation of GBS from blood culture or PCR or latex agglutination. |
| Meningitis | (1) Clinical signs of pSBI and isolation of GBS from cerebrospinal fluid or PCR or latex agglutination, or (2) Isolation of GBS from blood culture or PCR or latex agglutination and CSF leucocyte count of >20x10^6^/l. |

Abbreviations: GBS = group B Streptococcus; pSBI = possible serious bacterial infection; PCR = polymerase chain reaction; CSF = cerebrospinal fluid

Modified from the protocol paper

## Supplementary Table 3. Assessment tool Child Behaviour Checklist and applicable measurement scales

| Version | Problem scales | DSM-5 oriented scales |
| --- | --- | --- |
| CBCL/1.5-5 | Internalising(emotionally reactive, anxious/depressed, somatic complaints and withdrawn), externalising(attention problems and aggressive behaviour) and total problems | depressive, anxiety, autism spectrum, attention deficient/hyperactivity, and oppositional defiant problems |
| CBCL/6-18 | Internalising(anxious/depressed, withdrawn/depressed, somatic complaints), externalising(rule-breaking problems and aggressive behaviour) and total problems | depressive, anxiety, somatic, attention deficient/hyperactivity, oppositional defiant and conduct problems |

Abbreviations: CBCL = child behaviour checklist; DSM = the diagnostic and statistical manual of mental disorders

## Supplementary Table 4. Adjusted means for iGBS survivors and non-GBS group (N=553)

|  | South Africa | | Mozambique | | India | | Kenya | | Argentina | |
| --- | --- | --- | --- | --- | --- | --- | --- | --- | --- | --- |
|  | iGBS | Non-GBS | iGBS | Non-GBS | iGBS | Non-GBS | iGSB | Non-GBS | iGBS | Non-GBS |
| **Pre-school aged children** | | | | | | | | | | |
| Total problems | 28.5  (15.8, 41.0) | 31.7  (19.0, 44.4) | 0.2  (-7.6, 8.1) | 3.6  (-3.0, 10.1) | 20.5  (14.6, 26.5) | 23.9  (18.9, 28.8) | 25.3  (18.5, 32.0) | 28.6  (23.1, 34.2) | 34.4  (17.6, 51.2) | 37.7  (21.1, 54.3) |
| Externalizing problems | 8.9  (3.6, 14.2) | 9.9  (4.5, 15.3) | -0.2  (-3.5, 3.1) | 0.8  (-2.0, 3.5) | 9.3  (6.8, 11.8) | 10.3  (8.2, 12.4) | 7.1  (4.3, 10.0) | 8.1  (5.8, 10.5) | 15.7  (8.6, 22.7) | 16.6  (9.6, 23.7) |
| Internalizing problems | 7.8  (3.9, 11.7) | 8.7  (4.8, 12.6) | 0.4  (-2.0, 2.8) | 1.3  (-0.7, 3.4) | 5.2  (3.4, 7.0) | 6.3  (4.6, 7.7) | 8.6  (6.6, 10.7) | 9.6  (7.9, 11.3) | 7.5  (2.4, 12.7) | 8.5  (3.3, 13.6) |
| **School-aged children** | | | | | | | | | | |
| Total problems | 37.3  (33.0, 41.5) | 33.2  (29.8, 36.6) | 6.3  (1.8, 10.8) | 2.3  (-1.3, 5.9) | 23.8  (17.4, 30.2) | 19.8  (13.7, 25.9) | 17.3  (12.5, 22.1) | 13.3  (9.3, 17.3) | 36.8  (28.9, 44.6) | 32.7  (24.7, 40.8) |
| Externalizing problems | 9.6  (8.3, 10.8) | 8.5  (7.5, 9.5) | 1.34  (0.03, 2.6) | 0.3  (-0.7, 1.4) | 7.0  (5.2, 8.9) | 6.0  (4.2, 7.8) | 3.8  (2.4, 5.2) | 2.7  (1.6, 3.9) | 8.3  (6.0, 10.6) | 7.3  (4.9, 9.6) |
| Internalizing problems | 10.3  (8.9, 11.6) | 9.1  (8.0,10.2) | 2.1  (0.7, 3.6) | 0.99  (-0.1, 2.1) | 5.6  (3.6, 7.6) | 4.4  (2.5, 6.4) | 6.0  (4.5, 7.5) | 4.8  (3.6, 6.1) | 11.4  (8.9, 13.8) | 9.1  (8.1, 10.2) |

Data presented as adjusted mean(95% confidence interval)

## Supplementary Table 5. Proportion of clinically significant Child Behaviour Checklist problems in the preschool-aged and school-aged cohorts across five LMIC

|  | Preschool-aged cohort | | | School-aged cohort | | |
| --- | --- | --- | --- | --- | --- | --- |
|  | iGBS survivors  (n=54) | non-iGBS comparison group  (n=109) | P-value | iGBS survivors  (n=104) | non-iGBS comparison group  (n=286) | P-value |
| Total  problems | 2(3.7) | 8(7.3) | 0.36 | 13(12.5) | 29(10.1) | 0.51 |
| Externalizing  problems | 3(5.6) | 7(6.4) | 0.83 | 10(9.6) | 21(7.3) | 0.46 |
| Internalizing  problems | 3(5.6) | 6(5.5) | 0.99 | 17(16.4) | 32(11.2) | 0.17 |

Abbreviations: LMIC = Low-to-Middle-Income Country; iGBS = invasive GBS disease

Data presented as n/N(%)

## Supplementary Table 6A. Proportion of clinically significant Child Behaviour Checklist problems in the preschool-aged cohort in five LMIC

|  | India(n=69) | | Kenya(n=47) | | Mozambique(n=32) | | South Africa(n=10) | | Argentina(n=5) | |
| --- | --- | --- | --- | --- | --- | --- | --- | --- | --- | --- |
|  | iGBS Survivors  (pre=25) | non-iGBS comparison group (n=44) | iGBS Survivors  (n=14) | non-iGBS comparison group(n=33) | iGBS Survivors  (n=8) | non-iGBS comparison group (n=24) | iGBS Survivors  (n=5) | non-iGBS comparison group (n=5) | iGBS Survivors  (n=2) | non-iGBS comparison group(n=3) |
| Total  problems | 0(0.0) | 4(9.1) | 1(7.1) | 3(9.1) | 0(0.0) | 0(0.0) | 1(20.0) | 0(0.0) | 0(0.0) | 1(33.3) |
| Externalizing  problems | 2(8.0) | 5(11.4) | 0(0.0) | 1(3.0) | 0(0.0) | 0(0.0) | 1(20.0) | 0(0.0) | 0(0.0) | 1(33.3) |
| Internalizing  problems | 1(4.0) | 1(2.3) | 1(7.1) | 5(15.2) | 0(0.0) | 0(0.0) | 1(20.0) | 0(0.0) | 0(0.0) | 0(0.0) |

Abbreviations: LMIC = Low-to-Middle-Income Country; iGBS = invasive GBS disease

Data presented as n/N(%)

## Supplementary Table 6B. Proportion of clinically significant Child Behaviour Checklist problems in the school-aged cohort in five LMIC

|  | India(n=27) | | Kenya(n=66) | | Mozambique(n=131) | | South Africa(n=150) | | Argentina(n=16) | |
| --- | --- | --- | --- | --- | --- | --- | --- | --- | --- | --- |
|  | iGBS Survivors  (pre=10) | non-iGBS comparison group (n=17) | iGBS Survivors  (n=15) | non-iGBS comparison group(n=51) | iGBS Survivors  (n=31) | non-iGBS comparison group  (n=100) | iGBS Survivors  (n=38) | non-iGBS comparison group (n=112) | iGBS Survivors  (n=10) | non-iGBS comparison group(n=6) |
| Total  problems | 0(0.0) | 0(0.0) | 1(6.7) | 1(2.0) | 0(0.0) | 0(0.0) | 10(26.3) | 26(23.2) | 2(20.0) | 2(33.3) |
| Externalizing  problems | 0(0.0) | 0(0.0) | 1(6.7) | 1(2.0) | 0(0.0) | 0(0.0) | 9(23.7) | 20(17.9) | 0(0.0) | 0(0.0) |
| Internalizing  problems | 0(0.0) | 0(0.0) | 2(13.3) | 0(0.0) | 0(0.0) | 0(0.0) | 11(29.0) | 30(26.8) | 4(40.0) | 2(33.3) |

Abbreviations: LMIC = Low- and Middle-Income Country; iGBS = invasive GBS disease

Data presented as n/N(%)

## Supplementary Table 7. Sensitivity analysis: Mean differences of the CBCL problem scores, stratified by preschool-aged and school-aged cohort, excluding moderate and severe neurodevelopmental impairment, N=502

|  | Adjusted mean differences (95% CI) | p-value |
| --- | --- | --- |
| **Pre-school aged children**  **iGBS n=45      Non iGBS n=101** |  |  |
| **Total  problems** | -0.24(-6.33, 5.85) | 0.938 |
| **Externalizing problems** | -0.07(-2.64, 2.49) | 0.956 |
| Attention problems | -0.37(-1.04, 0.30) | 0.279 |
| Aggressive problems | 0.30(-1.82, 2.41) | 0.782 |
| **Internalizing problems** | -0.004(-1.95, 1.94) | 0.996 |
| Emotional reactive | 0.03(-0.59, 0.66) | 0.914 |
| Anxious/depressed | -0.30(-0.94, 0.34) | 0.356 |
| Withdrawn | -0.38(-1.08, 0.31) | 0.277 |
| Somatic | 0.65(0.08, 1.21) | 0.025 |
| **School-aged children**  **iGBS n=88      Non iGBS n=268** |  |  |
| **Total  problems** | 4.31(0.43, 8.19) | 0.029 |
| **Externalizing problems** | 1.09(-0.04, 2.23) | 0.058 |
| Rule breaking behaviour | 0.50(0.09, 0.90) | 0.016 |
| Aggressive | 0.60(-0.24, 1.43) | 0.161 |
| **Internalizing problems** | 1.23(0.01, 2.45) | 0.048 |
| Anxious/depressed | 0.39(-0.24, 1.01) | 0.224 |
| Withdrawn/depressed | 0.39(-0.01, 0.80) | 0.058 |
| Somatic | 0.45(-0.07, 0.97) | 0.090 |

# STROBE Statement—Checklist of items that should be included in reports of cohort studies

|  | Item No | Recommendation | Page No |
| --- | --- | --- | --- |
| **Title and abstract** | 1 | (*a*) Indicate the study’s design with a commonly used term in the title or the abstract | 1 |
|  |  | (*b*) Provide in the abstract an informative and balanced summary of what was done and what was found | 2 |
| Introduction | | | |
| Background/rationale | 2 | Explain the scientific background and rationale for the investigation being reported | 2 |
| Objectives | 3 | State specific objectives, including any prespecified hypotheses | 5 |
| Methods | | | |
| Study design | 4 | Present key elements of study design early in the paper | 6-7 |
| Setting | 5 | Describe the setting, locations, and relevant dates, including periods of recruitment, exposure, follow-up, and data collection | 6-7 |
| Participants | 6 | (*a*) Give the eligibility criteria, and the sources and methods of selection of participants. Describe methods of follow-up | 6-7 |
|  |  | (*b*)For matched studies, give matching criteria and number of exposed and unexposed |  |
| Variables | 7 | Clearly define all outcomes, exposures, predictors, potential confounders, and effect modifiers. Give diagnostic criteria, if applicable | 6-7 |
| Data sources/ measurement | 8* | For each variable of interest, give sources of data and details of methods of assessment (measurement). Describe comparability of assessment methods if there is more than one group | 6-7 |
| Bias | 9 | Describe any efforts to address potential sources of bias | 6-7 |
| Study size | 10 | Explain how the study size was arrived at |  |
| Quantitative variables | 11 | Explain how quantitative variables were handled in the analyses. If applicable, describe which groupings were chosen and why | 6-7 |
| Statistical methods | 12 | (*a*) Describe all statistical methods, including those used to control for confounding | 8 |
|  |  | (*b*) Describe any methods used to examine subgroups and interactions |  |
|  |  | (*c*) Explain how missing data were addressed |  |
|  |  | (*d*) If applicable, explain how loss to follow-up was addressed |  |
|  |  | (*e*) Describe any sensitivity analyses |  |
| Results | | |  |
| Participants | 13* | (a) Report numbers of individuals at each stage of study—eg numbers potentially eligible, examined for eligibility, confirmed eligible, included in the study, completing follow-up, and analysed | 8 |
|  |  | (b) Give reasons for non-participation at each stage |  |
|  |  | (c) Consider use of a flow diagram |  |
| Descriptive data | 14* | (a) Give characteristics of study participants (eg demographic, clinical, social) and information on exposures and potential confounders |  |
|  |  | (b) Indicate number of participants with missing data for each variable of interest |  |
|  |  | (c) Summarise follow-up time (eg, average and total amount) | 8 |
| Outcome data | 15* | Report numbers of outcome events or summary measures over time | 9 |

| Main results | 16 | (*a*) Give unadjusted estimates and, if applicable, confounder-adjusted estimates and their precision (eg, 95% confidence interval). Make clear which confounders were adjusted for and why they were included |  |
| --- | --- | --- | --- |
|  |  | (*b*) Report category boundaries when continuous variables were categorized |  |
|  |  | (*c*) If relevant, consider translating estimates of relative risk into absolute risk for a meaningful time period | 9 |
| Other analyses | 17 | Report other analyses done—eg analyses of subgroups and interactions, and sensitivity analyses | 9 |
| Discussion | | | |
| Key results | 18 | Summarise key results with reference to study objectives | 10 |
| Limitations | 19 | Discuss limitations of the study, taking into account sources of potential bias or imprecision. Discuss both direction and magnitude of any potential bias | 10-11 |
| Interpretation | 20 | Give a cautious overall interpretation of results considering objectives, limitations, multiplicity of analyses, results from similar studies, and other relevant evidence | 10-11 |
| Generalisability | 21 | Discuss the generalisability (external validity) of the study results | 11 |
| Other information | | | |
| Funding | 22 | Give the source of funding and the role of the funders for the present study and, if applicable, for the original study on which the present article is based | 14 |

*Give information separately for exposed and unexposed groups
